# A new transfer entropy method for measuring directed connectivity from complex-valued fMRI data

**DOI:** 10.3389/fnins.2024.1423014

**Published:** 2024-07-10

**Authors:** Wei-Xing Li, Qiu-Hua Lin, Chao-Ying Zhang, Yue Han, Vince D. Calhoun

**Affiliations:** ^1^School of Information and Communication Engineering, Dalian University of Technology, Dalian, China; ^2^Tri-Institutional Center for Translational Research in Neuroimaging and Data Science (TReNDS), Georgia State University, Georgia Institute of Technology, Emory University, Atlanta, GA, United States

**Keywords:** complex-valued fMRI data, transfer entropy, partial transfer entropy, directed connectivity, functional connectivity

## Abstract

**Background:**

Inferring directional connectivity of brain regions from functional magnetic resonance imaging (fMRI) data has been shown to provide additional insights into predicting mental disorders such as schizophrenia. However, existing research has focused on the magnitude data from complex-valued fMRI data without considering the informative phase data, thus ignoring potentially important information.

**Methods:**

We propose a new complex-valued transfer entropy (CTE) method to measure causal links among brain regions in complex-valued fMRI data. We use the transfer entropy to model a general non-linear magnitude–magnitude and phase–phase directed connectivity and utilize partial transfer entropy to measure the complementary phase and magnitude effects on magnitude–phase and phase–magnitude causality. We also define the significance of the causality based on a statistical test and the shuffling strategy of the two complex-valued signals.

**Results:**

Simulated results verified higher accuracy of CTE than four causal analysis methods, including a simplified complex-valued approach and three real-valued approaches. Using experimental fMRI data from schizophrenia and controls, CTE yields results consistent with previous findings but with more significant group differences. The proposed method detects new directed connectivity related to the right frontal parietal regions and achieves 10.2–20.9% higher SVM classification accuracy when inferring directed connectivity using anatomical automatic labeling (AAL) regions as features.

**Conclusion:**

The proposed CTE provides a new general method for fully detecting highly predictive directed connectivity from complex-valued fMRI data, with magnitude-only fMRI data as a specific case.

## 1 Introduction

To date, a huge number of studies have investigated directed functional connectivity (FC) or functional network connectivity (FNC) using fMRI data (Demirci et al., 2009; Stevens et al., 2009; Lizier et al., 2011; Ursino et al., 2020; Crimi et al., 2021). Directed FC/FNC refers to the statistical causality between different time series from brain regions of interest (ROIs) or time courses of brain networks extracted by data-driven methods from fMRI data (Stevens et al., 2009; Ursino et al., 2020; Mahmood et al., 2022). The directed FC/FNC results have been widely used as putative biomarkers to identify/predict brain function changes linked to mental disorders such as schizophrenia (Fogelson et al., 2014; Bastos-Leite et al., 2015; Dietz et al., 2020).

Directed FC/FNC analyses can be generally classified into model-based and model-free methods. Typical model-based methods include dynamic causal modeling (DCM) (Friston et al., 2019), structural equation modeling (Bielczyk et al., 2019), and dynamic Bayesian network (Wu et al., 2014). Regarding model-free methods, the Granger causal test is frequently used to determine whether there is a linear causal relationship between ROIs and brain networks (Demirci et al., 2009; Crimi et al., 2021). Demirci et al. (2009) exploited the Granger causal test to calculate directed FNC of fMRI data and found abnormal connections from frontal areas to visual areas for patients with schizophrenia. Crimi et al. (2021) used Granger connections to classify patients with autism spectrum disorder and healthy controls.

Real-valued transfer entropy is utilized to identify the underlying non-linearly directed information between ROIs or between brain networks (Lizier et al., 2011; Ursino et al., 2020; Liu et al., 2022). Ursino et al. (2020) verified that transfer entropy is a promising method to estimate the causality of connections between regions with long time delays. Lizier et al. (2011) presented a transfer entropy method to detect causality between brain regions in cognitive tasks and showed task difficulty being related to causal strength for the motor cortex. Following this, Liu et al. (2022) proposed a scored function based on transfer entropy and conditional entropy to quantify directed FC, which accurately inferred directed connectivity networks of time series. The most commonly used method for estimating real-valued transfer entropy is the histogram-based transfer entropy (HTE), which estimates the joint probability density function via a histogram-based function. Other transfer entropy algorithms were proposed to improve the accuracy of causal inference or noise robustness, including symbolic transfer entropy (STE) (Li and Zhang, 2022), effective transfer entropy (Behrendt et al., 2019; Caserini and Pagnottoni, 2022), Renyi transfer entropy (Jizba et al., 2022; Zhang et al., 2023), and phase transfer entropy (Wang and Chen, 2020; Gu et al., 2021).

Our study is motivated by two key points. First, previous studies show non-linear FC/FNC properties in fMRI (Li et al., 2010, 2011; Motlaghian et al., 2023). The transfer entropy approach is designed to forecast non-linear causality (Schreiber, 2000), while the Granger causal test may fail as a linear model-free approach (Bastos and Schoffelen, 2016). Second, fMRI data are initially acquired as complex-valued image pairs including both magnitude and phase data (Calhoun et al., 2002; Rowe and Logan, 2004; Adali and Calhoun, 2007). A new transfer entropy approach is needed to incorporate unique and additional information from the phase data in addition to the magnitude-only fMRI data (Yu et al., 2015). The simple sum of the separate real-valued results from the magnitude and the phase data suffers from a loss of accuracy as there is also a correlation between the magnitude and the phase. As such, we propose a new complex-valued transfer entropy (CTE) to detect full causality between two complex-valued signals.

The main contributions of this study are 3-fold:

1. We propose a new CTE method to measure non-linear causal (directed) connectivity among two complex-valued signals by incorporating complementary causality between magnitude and phase using the partial transfer entropy, in addition to detecting magnitude–magnitude and phase–phase causality using transfer entropy. Simulated data verify the high accuracy of CTE compared to a simplified CTE (sCTE) without magnitude–phase causality and the three real-valued methods, including STE, HTE, and Granger causal test.
2. We evaluate the significance of the non-linear directed connectivity via a one-sample *t*-test by using a shuffling strategy of two complex-valued signals. The statistical test assists in eliminating spurious causality, ensuring the stability and accuracy of the causality measurement.
3. We analyze directed FC using experimental resting-state complex-valued fMRI data from 40 schizophrenia patients and 40 healthy controls. CTE yields results consistent with previous findings but with more significant group differences, detects new directed connectivity, and achieves higher SVM classification accuracy, compared to sCTE, STE, HTE, and Granger causal test.

## 2 Methods

### 2.1 Modeling and deviation of CTE

**Figure 1:**
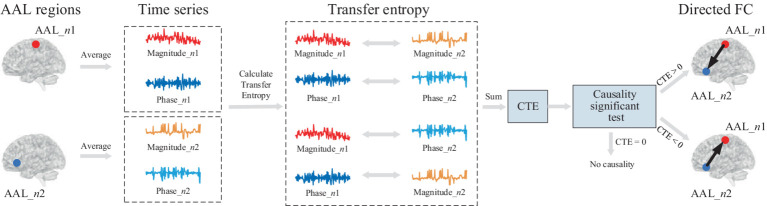
Framework diagram for directed FC measured by CTE. First, average complex-valued time series from two AAL regions are obtained. Each average time series involves magnitude and phase. To quantify complete complex-valued causality, CTE considers four parts of causality, including magnitude–magnitude, phase–phase, and two magnitude–phase causality. After the causal significant test, the directed FC between two regions is measured by CTE.

Figure 1 shows the framework diagram for measuring directed FC using CTE. Take two AAL regions AAL_*n*1 and AAL_*n*2 for example, each region can obtain an average complex-valued time series involving magnitude and phase. To quantify complete complex-valued causality, CTE measures magnitude–magnitude and phase–phase, and two parts of magnitude and phase causality. To guarantee the reliability of causality measurement, a causal significant test is performed. The direction of FC can be judged by the polarity of CTE. If the CTE value is positive, the direction FC points from AAL_*n*1 to AAL_*n*2; if the CTE value is negative, the direction is the opposite; if CTE equals zero, there is no directed FC between the two AAL regions.

We denote two complex-valued signals as $z_{1}=\left\{z_{1}\left(t\right)\right\}={\left[z_{1}\left(1\right),\ldots ,z_{1}\left(T\right)\right]}^{T}$, $z_{2}=\left\{z_{2}\left(t\right)\right\}={\left[z_{2}\left(1\right),\ldots ,z_{2}\left(T\right)\right]}^{T}$, t=1,…,T, and *T* is the data length. The two signals are represented with magnitude and phase in Eq. (1) as follows:


(1) $$\begin{array}{cc} z_{1} & =a\exp\left(j\theta \right)\\ z_{2} & =b\exp\left(j\varphi \right) \end{array}$$

where $a\in {\mathbb{R}}^{T}$ and $\theta \in {\mathbb{R}}^{T}$ are the magnitude and phase of $z_{1}$, and $b\in {\mathbb{R}}^{T}$ and $\varphi \in {\mathbb{R}}^{T}$ are the magnitude and phase of $z_{2}$.

Based on the relationship between the magnitude and phase of the brain networks (Yu et al., 2015), we propose a definition of CTE considering complete causality between two complex-valued signals. Motivated by the complex-valued mutual information introduced by Goebel et al. (2011), CTE combines the magnitude and phase to make causality inference and is represented as follows:


(2) $$\begin{array}{c} C_{z1\to z2}=R_{a\to b}+R_{\theta \to \varphi }+R_{a\to b|\theta }+R_{\theta \to \varphi |a} \end{array}$$

where $R_{a\to b}$ and $R_{\theta \to \varphi }$ are real-valued transfer entropy from the magnitude and the phase of the two signals, respectively. $R_{a\to b|\theta }$ and $R_{\theta \to \varphi |a}$ are partial transfer entropy (Papana et al., 2012), which extends transfer entropy to account for the presence of the third variable. We extend to quantify the complementary phase and magnitude effects on the causality $R_{a\to b}$ and $R_{\theta \to \varphi }.$

Real-valued transfer entropy $R_{a\to b}$ and $R_{\theta \to \varphi }$ in Eq. (2) can be calculated as follows (Schreiber, 2000):


(3) $$\begin{array}{c} \begin{array}{cc} R_{a\to b} & =\sum p\left(b_{t},b_{t-\tau },a_{t-\tau }\right)\log\frac{p\left(b_{t}|b_{t-\tau },a_{t-\tau }\right)}{p\left(b_{t}|b_{t-\tau }\right)}\\ R_{\theta \to \varphi } & =\sum p\left(\varphi_{t},\varphi_{t-\tau },\theta_{t-\tau }\right)\log\frac{p\left(\varphi_{t}|\varphi_{t-\tau },\theta_{t-\tau }\right)}{p\left(\varphi_{t}|\varphi_{t-\tau }\right)} \end{array} \end{array}$$

where a→b denotes causal direction from a to b, p(⋅) is a marginal probability density function, ${\;}^{"}p{\left(\cdot |\cdot \right)}^{"}$ represents a condition probability density function, τ is the parameter of time delay, and $a_{t-\tau }$ and $b_{t-\tau }$ are delayed a and b by τ time points. By calculating the Pearson correlation coefficient between the two signals with different time delays, the time delay corresponding to the maximum correlation coefficient is selected as the value of τ.

Real-valued partial transfer entropy $R_{a\to b|\theta }$ in Eq. (2) is determined as follows:


(4) $$\begin{array}{c} R_{a\to b|\theta }=-\sum p\left(b_{t}|b_{t-\tau },\theta_{t-\tau }\right)\log\left\{p\left(b_{t}|b_{t-\tau },\theta_{t-\tau }\right)\right\}\\ +\sum p\left(b_{t}|a_{t-\tau },b_{t-\tau },\theta_{t-\tau }\right)\log\left\{p\left(b_{t}|a_{t-\tau },b_{t-\tau },\theta_{t-\tau }\right)\right\} \end{array}$$

In Eq. (4), it can be observed that phase θ and magnitude a are jointly used to determine the causal direction to magnitude b. In other words, $R_{a\to b|\theta }$ incorporates magnitude–phase causality between θ and b by quantifying the complementary magnitude effects of a on the causality. Similarly, $R_{\theta \to \varphi |a}$ can be calculated as follows:


(5) $$\begin{array}{c} R_{\theta \to \varphi |a}=-\sum p\left(\varphi_{t}|a_{t-\tau },\varphi_{t-\tau }\right)\log\left\{p\left(\varphi_{t}|a_{t-\tau },\varphi_{t-\tau }\right)\right\}\\ +\sum p\left(\varphi_{t}|\theta_{t-\tau },\varphi_{t-\tau },a_{t-\tau }\right)\log\left\{p\left(\varphi_{t}|\theta_{t-\tau },\varphi_{t-\tau },a_{t-\tau }\right)\right\} \end{array}$$

In Eqs. (2)–(5), we need to estimate condition probability density functions and joint probability density functions. We represent the condition probability density function with joint probability density functions and then estimate joint probability density functions. Taking $p\left(b_{t}|b_{t-\tau },\theta_{t-\tau }\right)$ as an example, we have the following:


(6) $$\begin{array}{c} p\left(b_{t}|b_{t-\tau },\theta_{t-\tau }\right)=\frac{p\left(\theta_{t-\tau },b_{t-\tau },b_{t}\right)}{p\left(\theta_{t-\tau },b_{t-\tau }\right)} \end{array}$$

where $p\left(\theta_{t-\tau },b_{t-\tau },b_{t}\right)$ and $p\left(\theta_{t-\tau },b_{t-\tau }\right)$ are joint probability density functions. To estimate the joint probability density functions, we perform symbolic processing on each of the variables. The symbolic process helps to improve the noise robustness to traditional transfer entropy and helps capture more non-linear causality proved by the previous study (Gu et al., 2021). Taking the phase $\theta ={\left[\theta \left(1\right),\ldots ,\theta \left(T\right)\right]}^{T}$ as an example, where superscript “T” represents the matrix transpose, the symbolic θ(t), 1≤t≤T, denoted as $\theta^{*}\left(t\right)$ is computed as follows (Wessel et al., 2000):


(7) $$\begin{array}{c} \theta^{*}\left(t\right)=\{\begin{array}{l} \begin{array}{cc} \;0: & \mu_{p}<\theta \left(t\right)\leq \left(1+\beta \right)\mu_{p} \end{array}\\ \begin{array}{c} \begin{array}{cc} \;1: & \left(1+\beta \right)\mu_{p}<\theta \left(t\right)<\infty \end{array}\\ \begin{array}{c} \begin{array}{ll} \;2: & \left(1-\beta \right)\mu_{p}<\theta \left(t\right)\leq \mu_{p} \end{array}\\ \begin{array}{cc} 3: & 0\leq \theta \left(t\right)\leq \left(1-\beta \right)\mu_{p} \end{array} \end{array} \end{array} \end{array},if\;\theta \left(t\right)\geq 0 \end{array}$$

(8) $$\begin{array}{c} \theta^{*}\left(t\right)=\{\begin{array}{l} \begin{array}{cc} \;0: & \left(1+\beta \right)\mu_{n}\leq \theta \left(t\right)<\mu_{n} \end{array}\\ \begin{array}{c} \;\begin{array}{cc} 1: & -\infty <\theta \left(t\right)<\left(1+\beta \right)\mu_{n} \end{array}\\ \begin{array}{c} \begin{array}{ll} \;2: & \mu_{n}\leq \theta \left(t\right)<\left(1-\beta \right)\mu_{n} \end{array}\;\\ \begin{array}{cc} \;3: & \left(1-\beta \right)\mu_{n}\leq \theta \left(t\right)<0 \end{array}\; \end{array} \end{array} \end{array},if\;\theta \left(t\right)<0 \end{array}$$

where β is a control parameter and set to be 0.05 according to the previous study (Wessel et al., 2000), and $\mu_{p}$ and $\mu_{n}$ are the mean of positive and negative variables of θ, respectively. As such, we obtain the symbolic variable vector of θ as $\theta^{*}={\left[\theta^{*}\left(1\right),\ldots ,\theta^{*}\left(T\right)\right]}^{T}.$ For simplicity, superscript “*” is omitted. φ can be symbolized in the same way. Magnitude a, b is symbolized only using Eq. (7) with non-negative values.

Then, we exploit a histogram-based method to estimate the joint probability density function by counting the number of common elements in segmented bins between vectors. Take $p\left(\theta_{t-\tau },b_{t-\tau }\right)$ in Eq. (6), for example, $\theta_{t-\tau }$ is divided into $k_{\theta }$ equal bins with the bin index denoted as *i*, and $b_{t-\tau }$ is divided into $k_{\theta }$ equal bins with the bin index as *j*. Denoting the segmented bin of $\theta_{t-\tau }$ and $b_{t-\tau }$ as Δθ and Δb, the joint probability density function $p\left(\theta_{t-\tau },b_{t-\tau }\right)$ is estimated by counting the elements number of $\theta_{t-\tau }$ and $b_{t-\tau }$ within the segmented bin [Δθ,Δb]. The parameters of bin width Δθ and Δb are determined by the number of segmented bins and data length. For simplicity, the parameters Δθ or Δb are equal and can be selected as follows:


(9) $$\begin{array}{c} \Delta \theta =\frac{\max\left\{\left[\theta_{t-\tau };b_{t-\tau }\right]\right\}-\min\left\{\left[\theta_{t-\tau };b_{t-\tau }\right]\right\}}{T} \end{array}$$

Thus, the joint probability density function $p\left(\theta_{t-\tau },b_{t-\tau }\right)$ located around the point (i,j) is represented as follows:


(10) $$\begin{array}{c} p\left(\theta_{t-\tau },b_{t-\tau }\right)=\frac{num\left(i,j\right)}{T-\tau } \end{array}$$

where num(Δθ,Δb) is the number of elements between $\theta_{t-\tau }$ and $b_{t-\tau }$ within the segmented bin [Δθ,Δb] around (i,j).

### 2.2 Significance test of causality

The complex-valued transfer entropy $C_{z1\to z2}$ quantifies causality from $z_{1}$ to $z_{2}$ but cannot measure the significance of the causality. As such, we define the causality significance using a statistical test together with a shuffling strategy, which has been previously used in real-valued transfer entropy studies (Bossomaier et al., 2016). The shuffling process assists in eliminating spurious causality between $z_{1}$ and $z_{2}$, ensuring the stability and accuracy of the causality measurement. Various transfer entropy differences between the original and shuffled signals are obtained by repeating the shuffling process (*R* times). Here, the number of times we perform shuffling, i.e., *R*, is set to 100. Then, one-sample *t*-test on the *R* causality differences is performed to detect the causality significance from $z_{1}$ to $z_{2}$.

If we denote the shuffled transfer entropy as $C_{z1\to z2}^{\text{shuffled}}$, the transfer entropy difference $C_{z1\to z2}^{*}$ is obtained in Eq. (11) as follows:


(11) $$\begin{array}{c} C_{z1\to z2}^{*}=C_{z1\to z2}-C_{z1\to z2}^{\text{shuffled}} \end{array}$$

Similarly, $C_{z2\to z1}^{*}$ is obtained in Eq. (12) as follows:


(12) $$\begin{array}{c} C_{z2\to z1}^{*}=C_{z2\to z1}-C_{z2\to z1}^{\text{shuffled}} \end{array}$$

Let $\Delta C=C_{z1\to z2}^{*}-C_{z2\to z1}^{*},$ $\Delta C={\left[\Delta C^{\left(1\right)},\ldots ,\Delta C^{\left(R\right)}\right]}^{T}$ includes all the transfer entropy differences between $C_{z1\to z2}^{*}$ and $C_{z2\to z1}^{*}$, we perform one sample *t*-test with the false discovery rate (FDR) correction as follows (Guo and Bhaskara, 2008):


(13) $$\begin{array}{c} \Delta C=\{\begin{array}{cc} \Delta \bar{C} & p.\text{ttest}\left(\Delta C\right)<p_{\text{th}}\\ 0, & \text{otherwise} \end{array} \end{array}$$

where $\Delta \bar{C}$ is the mean of ΔC, $p_{\text{th}}$=0.05. We define the causal direction by the sign of ΔC as follows:


(14) $$\begin{array}{c} \text{sig}\_\text{causality}=\{\begin{array}{cc} z_{1}\to z_{2}, & \Delta C>0\\ z_{2}\to z_{1}, & \Delta C<0\\ \text{no}\;\text{causality}, & \Delta C=0 \end{array} \end{array}$$

## 3 Experimental methods

### 3.1 Simulated signals

To evaluate the efficacy of CTE, we generate two sets of simulated complex-valued signals with linear and non-linear causality, respectively. Each set has three types of causal directions and is randomly generated 1,000 times and divided into 10 groups.

The baseline signals are generated using a widely used MATLAB toolbox named Granger causal connectivity analysis (GCCA) (Seth, 2010). The signals are generated with real-valued linear causality via an AR model as Seth (2010):


(15) $$\begin{array}{c} \begin{array}{cc} x_{1}\left(t\right) & =0.95\sqrt{2}x_{1}\left(t-1\right)-0.9025x_{1}\left(t-2\right)+w_{1}\left(t\right)\\ x_{2}\left(t\right) & =0.5x_{1}\left(t-1\right)+w_{2}\left(t\right) \end{array} \end{array}$$

where 3≤t≤T, *T* is the data length and set to be 146 to keep the same data length as in the fMRI data. $w_{1}\left(t\right)$ and $w_{2}\left(t\right)$ are random variables with zero mean and unit variance satisfying normal distribution. The linear and non-linear causality with different causality cases can be obtained by exploiting and modifying the baseline signals defined in Eq. (15).

When generating simulated signals with linear causality, the three types of simulated complex-valued signals are denoted as type L1, L2, and L3, respectively. The magnitude and phase of the two signals $z_{1}$ and $z_{2}$ from the three linear types are generated using Eqs. (16)–(18) as follows::

1.typeL1:


(16) $$\begin{array}{c} \begin{array}{cc} a\left(t\right) & =0.95\sqrt{2}a\left(t-1\right)-0.9025a\left(t-2\right)+w_{1}\left(t\right)\\ b\left(t\right) & =0.5a\left(t-1\right)+w_{2}\left(t\right)\\ \begin{array}{c} \theta \left(t\right)\\ \varphi \left(t\right) \end{array} & \begin{array}{l} =0.95a\left(t\right)-0.9025a\left(t-2\right)+w_{1}\left(t\right)\\ =-0.6a\left(t\right)+w_{2}\left(t\right) \end{array} \end{array} \end{array}$$

2. typeL2:


(17) $$\begin{array}{c} \begin{array}{cc} a\left(t\right) & =0.95\sqrt{2}a\left(t-1\right)-0.9025a\left(t-2\right)+w_{1}\left(t\right)\\ b\left(t\right) & =0.5a\left(t-1\right)+w_{2}\left(t\right)\\ \begin{array}{c} \theta \left(t\right)\\ \varphi \left(t\right) \end{array} & \begin{array}{l} =0.95\theta \left(t\right)-0.9025\theta \left(t-2\right)+w_{3}\left(t\right)\\ =-0.6\theta \left(t\right)+w_{4}\left(t\right) \end{array} \end{array} \end{array}$$

3. typeL3:


(18) $$\begin{array}{c} \begin{array}{cc} a\left(t\right) & =0.95\sqrt{2}a\left(t-1\right)-0.9025a\left(t-2\right)+w_{1}\left(t\right)\\ b\left(t\right) & =0.5a\left(t-1\right)+w_{2}\left(t\right)\\ \begin{array}{c} \theta \left(t\right)\\ \varphi \left(t\right) \end{array} & \begin{array}{l} =r_{1}\left(t\right)\\ =r_{2}\left(t\right) \end{array} \end{array} \end{array}$$

where $r_{1}\left(t\right)$ and $r_{2}\left(t\right)$ are randoms without causality.

The non-linear causality can be obtained by adding quadratic and three-order terms to (Eq. 15). The magnitude and phase of the three types (N1, N2, and N3) can be generated using Eqs. (19)–(21) as follows:

1. typeN1:


(19) $$\begin{array}{c} \begin{array}{cc} a\left(t\right) & =0.95\sqrt{2}a\left(t-1\right)-0.9025a\left(t-2\right)+w_{1}\left(t\right)\\ b\left(t\right) & =0.5a^{2}\left(t-1\right)+w_{2}\left(t\right)\\ \begin{array}{c} \theta \left(t\right)\\ \varphi \left(t\right) \end{array} & \begin{array}{l} =0.95a\left(t\right)-0.9025a\left(t-2\right)+w_{1}\left(t\right)\\ =-0.6a^{3}\left(t\right)+w_{2}\left(t\right) \end{array} \end{array} \end{array}$$

2.typeN2:


(20) $$\begin{array}{c} \begin{array}{cc} a\left(t\right) & =0.95\sqrt{2}a\left(t-1\right)-0.9025a\left(t-2\right)+w_{1}\left(t\right)\\ b\left(t\right) & =0.5a^{2}\left(t-1\right)+w_{2}\left(t\right)\\ \begin{array}{c} \theta \left(t\right)\\ \varphi \left(t\right) \end{array} & \begin{array}{l} =0.95\theta \left(t\right)-0.9025\theta \left(t-2\right)+w_{3}\left(t\right)\\ =-0.6\theta^{3}\left(t\right)+w_{4}\left(t\right) \end{array} \end{array} \end{array}$$

3.typeN3:


(21) $$\begin{array}{c} \begin{array}{cc} a\left(t\right) & =0.95\sqrt{2}a\left(t-1\right)-0.9025a\left(t-2\right)+w_{1}\left(t\right)\\ b\left(t\right) & =0.5a^{2}\left(t-1\right)+w_{2}\left(t\right)\\ \begin{array}{c} \theta \left(t\right)\\ \varphi \left(t\right) \end{array} & \begin{array}{l} =r{\;}_{1}\left(t\right)\\ =r_{2}\left(t\right) \end{array} \end{array} \end{array}$$

where $w_{1}\left(t\right)$, $w_{2}\left(t\right)$, $w_{3}\left(t\right),$ and $w_{4}\left(t\right)$ are variables with zero-valued mean, unit variance satisfying normal distribution, and without causality.

**Figure 2:**
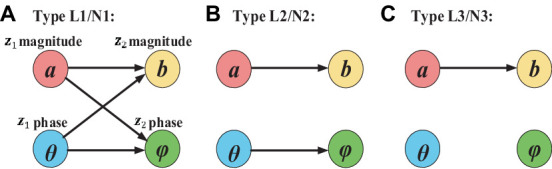
Ground truth casual direction for three types of simulated linear and non-linear signals. The arrow represents the causal direction.

Figure 2 shows the ground truth causal directions for the three types of two simulated complex-valued signals with non-linear and linear causality. Specifically, type L1/N1 has the complete complex-valued causality including magnitude–magnitude, phase–phase, and magnitude–phase; type L2/N2 has the incomplete complex-valued causality including magnitude–magnitude and phase–phase; type L3/N3 only has magnitude–magnitude causality.

**Figure 3:**
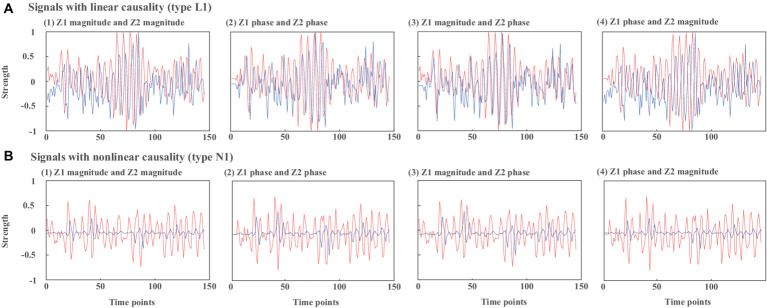
Waveforms of simulated signals z1 and z2 from **(A)** type L1 and **(B)** type N1. (1) z1 magnitude and z2 magnitude, (2) z1 phase and z2 phase, (3) z1 magnitude and z2 phase, (4) z1 phase and z2 magnitude.

Figure 3 presents example waveforms of simulated signals z_1_ and z_2_ from type L1 and type N1. The ground-truth causal direction for the magnitude and phase is shown in Figure 2A. We observe the peaks of the cause signals (z_1_ magnitude and z_1_ phase, in red) are ahead of the effect signals (z_2_ magnitude and z_2_ phase, in blue) in all cases, which are consistent with the causal direction of Figure 2. To test the noise effects on CTE, we also add Gaussian noise to the simulated signals with the signal-to-noise ratio (SNR) ranging from −10 dB to 10 dB.

### 3.2 Experimental fMRI data

The resting-state complex-valued fMRI data were a self-collected dataset from 80 subjects, including 40 healthy controls (HCs) and 40 patients with schizophrenia (SZs) with written subject consent overseen by the University of New Mexico Institutional Review Board. Specifically, there are 28 men and 12 women for HCs (mean age ± standard deviation: 36.25 ± 11.40) and 33 men and 7 women for SZs (mean age ± standard deviation: 40.73 ± 14.43). During the scan, all the participants were instructed to rest quietly in the scanner and keep their eyes open without sleeping and not to think of anything in particular (Lin et al., 2022). fMRI scans were acquired by a Siemens 3 T TIM Trio scanner equipped with a 12-channel head coil. The functional scan was acquired with the following parameters: TR = 2 s, TE = 29 ms, field of view = 24 cm, acquisition matrix = 64 × 64, flip angle = 75°, slice thickness = 3.5 mm, and slice gap = 1 mm. Data preprocessing was performed using the SPM software package.^1^ Functional images were motion-corrected and then spatially normalized into the standard Montreal Neurological Institute space. Following spatial normalization, the data were resampled to 3 × 3 × 3 mm^3^, resulting in 53 × 63 × 46 voxels. Both magnitude and phase images were spatially smoothed with an 8 × 8 × 8 mm^3^ full-width half-maximum (FWHM) Gaussian kernel. Phase images were first motion corrected using the transformations computed from magnitude-only data; then, complex division of phase data by the first time point reduced the need for phase unwrapping; and spatial normalization of phase images used the warp parameters computed from magnitude-only data.

### 3.3 Complex-valued time series of ROI

Brodmann area (BA) and anatomical automatic labeling (AAL) atlas are two commonly used references to divide the brain into ROIs for FC analysis. Compared with BA, AAL obtains more ROIs and involves the cerebellum regions. To achieve a more comprehensive and detailed segmentation of the brain regions, we used AAL to obtain 116 ROIs (Tzourio-Mazoyer et al., 2002) and divided the 116 ROIs into 10 brain networks proposed by Smith et al. (2009), consisting of medial visual areas (MV), occipital pole visual areas (OPV), lateral visual areas (LV), default mode network (DMN), cerebellum (CER), sensorimotor (SEM), temporal lobe (TEM), anterior DMN (ADMN), left frontal parietal area (LFP), and right frontal parietal area (RFP). By dividing the 116 ROIs into these 10 networks, it is better to reveal the regularities of connections and establish relationships between FC and FNC.

The complex-valued time series for each ROI is expressed in Eq. (22) as follows:


(22) $$\begin{array}{c} x_{n}=\left|{\bar{x}}_{n}\left(t\right)\right|\exp\left\{j\varphi \left({\bar{x}}_{n}\left(t\right)\right)\right\} \end{array}$$

where $\left|{\bar{x}}_{n}\left(t\right)\right|$$\varphi \left({\bar{x}}_{n}\left(t\right)\right),n=1,..,116,t=1,..,T$ are the averaged magnitude and phase time series across all voxels within each ROI, and *T* denotes the total number of time points. The causality between any two ROIs can be quantified by CTE as $\Delta C\left\{x_{n1},x_{n2}\right\}$ using Eqs. (2), (13), and (14).

### 3.4 Performance measures

In order to evaluate the proposed CTE, we compare it with the three real-valued causal analysis methods STE, HTE, and Granger, and one complex-valued approach, i.e., sCTE without considering magnitude and phase causality defined in Eq. (23) as follows:


(23) $$\begin{array}{c} {\tilde{C}}_{z1\to z2}=R_{a\to b}+R_{\theta \to \varphi } \end{array}$$

For the real-valued causal methods, both STE and HTE calculate real-valued TE $R_{a\to b}$ between magnitudes in Eq. (3). Specifically, STE utilized the symbolic process in Eqs. (7) and (8) before estimating joint PDF using Eqs. (9) and (10), while HTE estimates joint PDF without symbolic process.

Granger causal test is based on utilizing linear regression models to perform a statistical causality inference. Given two variables a and b, the autoregressive (AR) model of the Granger causal test is represented in Eq. (24) as follows:


(24) $$\begin{array}{c} \begin{array}{c} a_{t}=\sum_{j=1}^{J}u_{j}a_{t-j}+\sum_{j=1}^{J}v_{j}b_{t-j}+\varepsilon_{t}\\ a_{t}=\sum_{j=1}^{J}c_{j}a_{t-j}+\eta_{t}\; \end{array} \end{array}$$

where $u_{j}$, $v_{j}$, and $c_{j}$ are the regression coefficients for the model, *J* is the estimated time delay between a and b, and $\varepsilon_{t}$ and $\eta_{t}$ are two independent series satisfying Gaussian distribution. The fitting variances of using $a_{t-j}$, $b_{t-j}$ to fit $a_{t}$, and only using $a_{t-j}$ are denoted as $\sigma^{2}\left(a_{t}|,a_{t-j}|,b_{t-j}\right)$ and $\sigma^{2}\left(a_{t}|a_{t-j}\right)$, respectively. The causal direction between a and b is judged by comparing the fitting variance using Eq. (25) as follows:


(25) $$\begin{array}{c} b\to a,if\;\sigma^{2}\left(a_{t}|a_{t-j}\right)>\sigma^{2}\left(a_{t}|,a_{t-j}|,b_{t-j}\right) \end{array}$$

As such, the causal direction is evaluated by Granger causality.

For simulated signals, we calculate the accuracy of directed inference in Eq. (26), denoted as AOC, as follows:


(26) $$\begin{array}{c} \text{AOC}=N_{\text{correct}}/N_{\text{total}} \end{array}$$

where $N_{\text{correct}}$ is the number of correct causal direction judgments and $N_{\text{total}}$ is the total number of causality evaluations between two signals.

For experimental fMRI data, we first calculate the average Pearson correlation coefficient between the magnitude and phase from two different ROI signals in HCs or SZs, to validate magnitude and phase dependence in Eq. (27) as follows:


(27) $$\begin{array}{c} \bar{\rho }=\frac{1}{K}\sum_{k=1}^{K}\text{cor}\left(\left|{\bar{x}}_{m}^{\left(k\right)}\right|,\varphi \left({\bar{x}}_{n}^{\left(k\right)}\right)\right),m,n=1,\ldots ,116 \end{array}$$

Second, we perform two-sample *t-tests* (*p*_th_ = 0.05) on connections from HCs and SZs with the FDR correction (Guo and Bhaskara, 2008) to obtain significant intergroup differences in Eq. (28) as follows:


(28) $$\begin{array}{c} d_{n,m}=\{\begin{array}{cc} t\_\text{value} & p.\text{ttest}2\left(\Delta C_{n,m}^{\left(\text{HC}\right)},\Delta C_{n,m}^{\left(\text{SZ}\right)}\right)<p_{\text{th}}\\ 0, & \text{otherwise} \end{array} \end{array}$$

where *n* and *m* represent two different ROIs or two brain networks, $\Delta C_{n,m}^{\left(\text{HC}\right)}=\left[\Delta C_{n,m}^{\left(\text{HC},1\right)},\ldots ,\Delta C_{n,m}^{\left(\text{HC},K\right)}\right]$ and $\Delta C_{n,m}^{\left(\text{SZ}\right)}=\left[\Delta C_{n,m}^{\left(\text{SZ},1\right)},\ldots ,\Delta C_{n,m}^{\left(\text{SZ},K\right)}\right].$

Third, we compare the number of common and unique connections detected by each method. Finally, we compare the efficacy of the common and unique connections as features to classify HCs and SZs using support vector machine (SVM). The multilayer perceptron kernel is selected, and SVM is repeated 1,000 times. Given a training dataset of K1 subjects as $\left[\left(x_{1},y_{1}\right),\ldots ,\left(x_{K1},y_{K1}\right)\right]$, where $x_{k}\in {\mathbb{C}}^{M1},1\leq k\leq K1$ represents the connectivity vector from the *k*th subject, *M*1 is the vector length, and $y_{k}$ is the label denoted as either 1 or − 1, indicating which class of $x_{k}$ belongs to. SVM aims to find a hyperplane to maximize the distance between the dataset and the hyperplane. The hyperplane can be represented in Eq. (29) as follows:


(29) $$\begin{array}{c} f\left(x_{k}\right)=\omega^{T}\phi \left(x_{k}\right)+b \end{array}$$

where ω and b are parameters of the hyperplane, and $\phi \left(x_{k}\right)$ is the kernel function. Multiple kernel functions can be used, e.g., the linear kernel, quadric kernel, and sigmoid kernel. By comparing the clustering performance, we select the multilayer perceptron (MLP) kernel for SVM and there are three layers including the input, hidden, and output layers. The input is the connectivity vectors $x_{k}$, the non-linear activation function is tanh{⋅}, and the output of the MLP kernel is represented in Eq. (30) as follows (Suykens and Vandewalle, 1999):


(30) $$\begin{array}{c} \phi \left(x_{k}\right)=\tanh\left\{\omega 1\cdot {\left(x_{k}\right)}^{T}\cdot x_{k}+b1\right\} \end{array}$$

where ω1 and b1 are weights and biases and are initially set to be 1 and −1, respectively. As such, the SVM classifier is built based on MLP kernel and can be realized by MATLAB built-in function named “mlp_kernel.”

The results are evaluated in terms of accuracy (ACC), sensitivity (SEN), and specificity (SPEC) defined in Eq. (31) as follows (Lin et al., 2022):


(31) $$\begin{array}{c} \begin{array}{l} \text{ACC}=\frac{\text{TP}+\text{TN}}{\text{TP}+\text{TN}+\text{FP}+\text{FN}}\\ \text{SEN}=\frac{\text{TP}}{\text{TP}+\text{FN}}\\ \text{SPEC}=\frac{\text{TN}}{\text{TN}+\text{FP}} \end{array} \end{array}$$

where TP, TN, FP, and FN denote true positive, true negative, false positive, and false negative, respectively. To mitigate overfitting and guarantee reliability, leave one out cross-validation (LOOV) is performed. Specifically, LOOV leaves out the data from one subject as test data and exploits the data from the rest of the selected subjects for training. Given that, the test data are independent of the training data in each LOOV loop. LOOV is used for cross-validation purposes, given we have limited data. As such, we repeat the validation 1,000 times.

## 4 Results

### 4.1 Simulated signals

Table 1 shows the accuracy of linear/non-linear directed inference for the three types of simulated signals without noise. Five types of directed analysis methods are compared including the proposed CTE, sCTE, and three real-valued methods: STE, HTE, and Granger. Compared with the other three transfer entropy methods (sCTE, STE, and THE), CTE obtains higher accuracy for causality inference, especially when having complete complex-valued causality (type L1/N1). Specifically, for type N1 (non-linear signals containing complete complex-valued causality), CTE achieves slightly higher accuracy than the sCTE and 18.7–85.9% higher accuracy than the three real-valued algorithms.

**Table 1:** Comparison of the mean and standard deviation of the accuracy of causality inference by five methods for simulated signals without noise.

		CTE (%)	sCTE (%)	STE (%)	HTE (%)	Granger (%)
Linear	Type L1	94.1 ± 1.5	88.4 ± 2.5	84.3 ± 4.7	52.7 ± 3.9	86.8 ± 1.7
	Type L2	89.2 ± 1.7	87.7 ± 2.8	83.5 ± 4.3	51.9 ± 4.3	89.1 ± 2.4
	Type L3	86.3 ± 3.3	85.8 ± 2.9	82.9 ± 4.1	50.3 ± 4.5	86.1 ± 2.6
Non-linear	Type N1	95.3 ± 1.3	87.5 ± 2.8	76.6 ± 3.5	66.1 ± 4.7	9.4 ± 2.9
	Type N2	91.3 ± 1.1	86.8 ± 2.6	74.2 ± 3.9	65.7 ± 4.4	8.1 ± 2.1
	Type N3	85.4 ± 3.7	83.3 ± 3.1	74.1 ± 4.1	64.3 ± 4.2	8.5 ± 2.6

**Figure 4:**
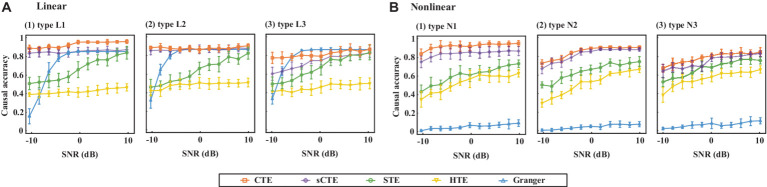
Causality accuracy for simulated signals with linear and non-linear causality under different SNRs.

Figure 4 shows the estimated causality accuracy for simulated signals with different SNRs. CTE achieves the highest accuracy and noise robustness for type L1/N1, due to the consideration of complete complex-valued causality. For type L2/N2, CTE and sCTE yield higher accuracy than three real-valued methods, including STE, HTE, and Granger, especially with low SNR (<-6 dB), due to the inclusion of phase causality. Regarding type L3/N3, CTE and the other transfer entropy algorithms have similar accuracy with high SNR (> 6 dB) as there only has magnitude causality. In this case, CTE is a general method suitable for measuring linear and non-linear causality for both complex-valued and real-valued signals. For type L3, note that Granger shows higher directed accuracy than CTE with SNR being -4 dB–4 dB. The reason is that Granger is built on the AR model for linear causality, making it optimal when only magnitude causality exists. However, Granger fails to detect non-linear causality. Therefore, considering both linear and non-linear scenarios, the proposed CTE is the optimal-directed algorithm in most cases.

### 4.2 Experimental fMRI data

After performing a two-sample *t*-test (*p* < 0.05, df = 78, FDR corrected) for the connections between HCs and SZs, we compare the numbers of common and unique connections detected by two different methods with significant HC-SZ differences. We select sCTE and STE as comparison methods since they have better performance for the simulated signals (refer to Figure 4).

**Figure 5:**
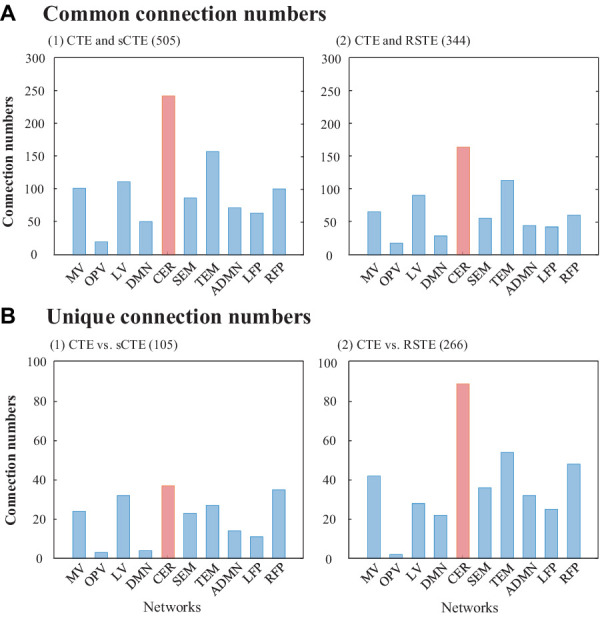
Common and unique connections between CTE and sCTE and between CTE and STE. The network with the maximum number of connections is highlighted in red.

Figure 5 shows the number of common and unique connections in terms of ten brain networks. In total, CTE obtains more common connections with sCTE than with STE (505 vs. 344), while detecting fewer unique connections with sCTE than with STE (105 vs. 266). The reason is that sCTE is closer to CTE by considering additional phase–phase causality relative to STE. Most of the common and unique connections belong to CER, which has been reported by previous studies to identify schizophrenia (Su et al., 2013; Watanabe et al., 2014). Other biomarker regions such as TEM, RFP, and visual areas (LV and MV) also show larger numbers of common and unique connections.

Table 2 shows common and unique connections between CTE and sCTE/STE with the top five significant HCs-SZs differences. These connections are mainly related to the brain networks including CER, RFP, and TEM, which are consistent with the abnormal connections of schizophrenia obtained by previous studies (Su et al., 2013; Watanabe et al., 2014; Oestreich et al., 2016; Maher et al., 2019; Dietz et al., 2020; Rashidi et al., 2021). Moreover, CTE detects unique connections with highly significant HCs-SZs differences related to RFP (vs. sCTE), DMN, and TEM (vs. STE). Considering the numbers and *t*-values of the connections with significant intergroup differences in Figure 5 and Table 2, CER, TEM, and RFP may be regarded as the biomarker brain networks for identifying schizophrenia (Su et al., 2013; Nenadic et al., 2014; Watanabe et al., 2014; Oestreich et al., 2016; Zhuo et al., 2018; Rashidi et al., 2021; Sklar et al., 2021).

**Table 2:** Common and unique connections with the top five HCs-SZs significance.

	Top five significant connections (AAL)	Networks	*p*-values (CTE)
Common (CTE and sCTE)	(1) No.64-No.100	RFP, CER	5.50 × 10^−5^
	(2) No.30-No.80	TEM	6.42 × 10^−5^
	(3) No.52-No.100	MV, CER	1.76 × 10^−4^
	(4) No.3-No.20	ADMN, SEM	4.59 × 10^−4^
	(5) No.92-No.111	CER	4.98 × 10^−4^
Common (CTE and STE)	(1) No.64-No.100	RFP, CER	5.50 × 10^−5^
	(2) No.52-No.100	MV, CER	1.76 × 10^−4^
	(3) No.80-No.100	TEM, CER	2.73 × 10^−4^
	(4) No.67-No.92	DMN, CER	5.59 × 10^−4^
	(5) No.18-No.92	TEM, CER	6.33 × 10^−4^
Unique (CTE vs. sCTE)	(1) No.14-No.28	RFP, LFP	0.0147
	(2) No.56-No.86	CER, LV	0.0167
	(3) No.37-No.64	MV, RFP	0.0173
	(4) No.20-No.102	SEM, CER	0.0178
	(5) No.66-No.78	RFP, OPV	0.0178
Unique (CTE vs. STE)	(1) No.25-No.29	DMN, TEM	0.0022
	(2) No.27-No.109	DMN, CER	0.0027
	(3) No.16-No.103	RFP, CER	0.0033
	(4) No.108-No.113	CER	0.0033
	(5) No.20-No.100	SEM, CER	0.0036

**Figure 6:**
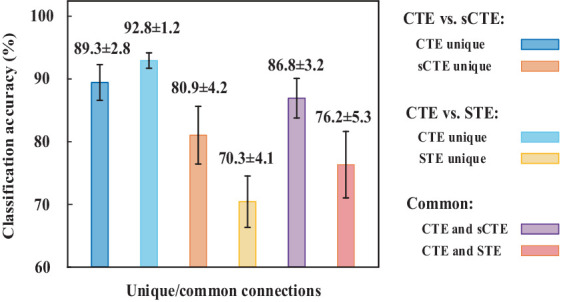
SVM classification accuracy for using unique/common connections obtained by CTE, sCTE, and STE, respectively. Unique connections of CTE achieve higher accuracy than others.

Figure 6 shows the SVM classification accuracy. The features are the unique or common connections obtained by CTE, sCTE, and STE. CTE exhibits the highest accuracy (92.8%) using unique connections relative to sCTE, followed by STE. As these unique connections of CTE are mainly related to RFP and CER shown in Table 2, it suggests that RFP- and CER-related connections helps to classify HCs and SZs. After verifying CTE unique connections are helpful in classification, exploiting all the significant connections including common and unique connections for classification is evaluated in Table 3.

**Table 3:** SVM classification is performed by combining unique connections and common connections.

	ACC (%)	SEN (%)	SPEC (%)
CTE	95.5 ± 2.8	96.3 ± 1.4	94.7 ± 5.1
sCTE	85.3 ± 7.2	90.5 ± 11.9	80.1 ± 9.6
STE	81.9 ± 10.1	85.0 ± 13.4	78.8 ± 12.3
HTE	76.8 ± 9.7	83.2 ± 11.2	70.4 ± 8.7
Granger	74.6 ± 8.6	77.1 ± 15.7	72.1 ± 10.9

For all the connections with significant intergroup differences, Table 3 shows the SVM performance measures (ACC, SEN, and SPEC) from five causality algorithms. As expected, the proposed complex-valued transfer entropy methods (CTE and sCTE) achieve better performance than real-valued directed analysis methods. CTE shows the best classification performance among all the five directed analysis methods; e.g., it improves higher ACC with 10.2% (95.5% vs. 85.3%) to sCTE, 13.6% (95.5% vs. 81.9%) to STE, 18.7% (95.5% vs. 76.8%) to HTE, and 20.9% (95.5% vs. 74.6%) to Granger, respectively. The proposed CTE obtains all the highest values of the three classification measures, especially for SEN reaching to 96.3%. This suggests that CTE captures meaningful and discriminative features to identify HCs and SZ.

Several studies have employed SVM for classifying HCs and SZs, especially using FC as features. In terms of using SVM for HCs and SZs classification, we select the previous studies with similar data sizes of the dataset in the paper (40 HCs and 40 SZs) for comparison. Su et al. (2013) performed SVM to FC quantified by an extended maximal information coefficient and obtained 82.8% clustering accuracy (32 HCs and 32 SZs). By analyzing the coherence regional homogeneity value, Liu et al. (2018) demonstrated that the abnormal connections related to TEM, insula, precentral gyrus, and precuneus can be used as psychosis biomarker of schizophrenia and achieved 89.9% accuracy (31 HCs and 48 SZs). Following this, Bae et al. (2018) pointed to decreased connections in the global and local network connectivity in SZs compared with HCs, especially in DMN, left parietal region, and TEM with an accuracy of 92.1% (31 HCs and 48 SZs). Instead of using FC of magnitude data for classification, Li et al. (2024) utilized dynamic connectivity features of phase maps as features for classification and obtained 87.5% accuracy (24 HCs and 24 SZs). As mentioned above, the existing studies of real-valued connections achieved 82.8–92.1% SVM accuracy for classifying HCs and SZs. Due to making full use of both magnitude and phase fMRI data, directed FC quantified by CTE shows higher classifying accuracy (95.5%) than the previous studies with similar data sizes.

## 5 Discussion

To our knowledge, few studies have explored directed FC based on complex-valued fMRI data, although directed FC has been increasingly studied using magnitude-only fMRI data. In this study, we propose a non-linear complex-valued directed analysis method based on transfer entropy to make full use of complex-valued fMRI data in highlighting differences between HCs and SZs. Simulated results show that our method has the highest accuracy and noisy robustness, especially for the non-linear model with complete complex-valued causality containing magnitude–magnitude, phase–phase, and magnitude–phase relationship. Experimental results show that CTE detects more unique connections with higher significant intergroup differences, thus leading to better performance in classifying HCs and SZs.

Instead of directly quantifying magnitude–phase causality, we propose to introduce partial transfer entropy to exploit the complementary phase/magnitude effects on magnitude–phase and phase–magnitude causality. This is because partial CTE can simultaneously utilize both magnitude and phase to assess causality in Eqs. (4) and (5), while transfer entropy only considers magnitude–phase or phase–magnitude dependence without the complementary phase/magnitude effects in Eqs. (32) and (33) as follows:


(32) $$\begin{array}{c} R_{b\to \theta }=\sum p\left(\theta ,\theta_{t-\tau },b_{t-\tau }\right)\log\frac{p\left(\theta_{t}|\theta_{t-\tau },b_{t-\tau }\right)}{p\left(\theta_{t}|\theta_{t-\tau }\right)} \end{array}$$

(33) $$\begin{array}{c} R_{a\to \varphi }=\sum p\left(\varphi_{t},\varphi_{t-\tau },a_{t-\tau }\right)\log\frac{p\left(\varphi_{t}|\varphi_{t-\tau },a_{t-\tau }\right)}{p\left(\varphi_{t}|\varphi_{t-\tau }\right)} \end{array}$$

As such, we use transfer entropy $R_{b\to \theta }$ and $R_{a\to \varphi }$ to replace partial entropy $R_{a\to b|\theta }$ and $R_{\theta \to \varphi |a}$ for comparison when calculating the proposed CTE.

**Figure 7:**
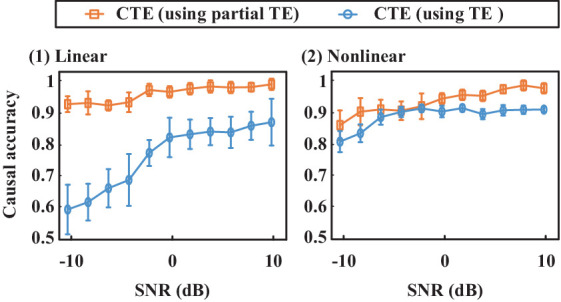
Causal inference accuracy comparison for CTE that using partial transfer entropy (shorted as partial TE) and TE, respectively. The proposed CTE using partial TE shows higher accuracy than the CTE that uses TE to directly quantify magnitude–phase causality.

Figure 7 shows directed accuracy for the simulated signals with linear and non-linear complete complex-valued causality (type L1 and N1). It presents that using partial transfer entropy (shorted as partial TE) to measure the complementary phase and magnitude effects on magnitude–phase causality shows better performance than those directly quantifying magnitude–phase causality using transfer entropy (TE), especially for quantifying the linear causality. When measuring the causality between magnitude and phase, partial TE considers more information, thus partial TE enhancing CTE noise robustness. It verifies the effectiveness of introducing partial TE in the proposed CTE definition.

To evaluate the data length effects on CTE, we change the simulated data length from 100 to 1,000 time points. CTE can keep high causality inference accuracy, especially for the signals with complete complex-valued causality (type L1 and N1). Because CTE keeps high causality inference accuracy to different data lengths, we can combine CTE and a sliding window approach for dynamic analysis. By performing causality analysis on the segmented time series, directed FC from different windows can be obtained. As such, dynamic statistical analysis can be exploited to analyze dynamics from the directed FC.

**Figure 8:**
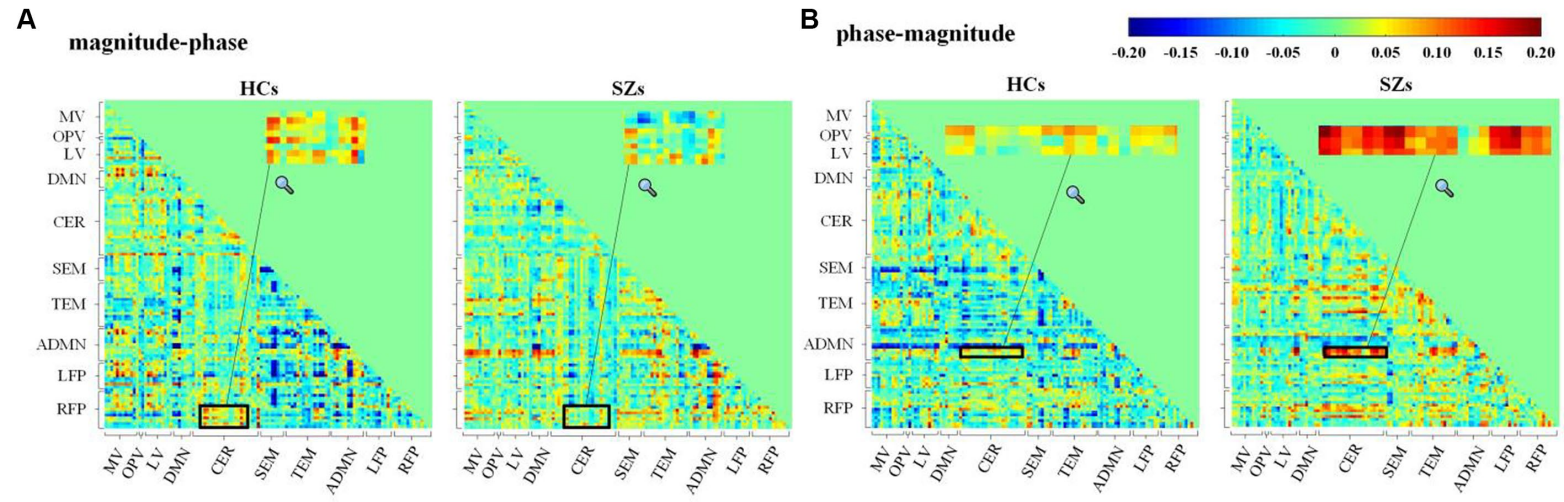
Average Pearson’s correlation coefficients for **(A)** magnitude–phase and **(B)** phase–magnitude of complex-valued time series from 116 AAL ROIs across subjects in HCs or SZs.

Figure 8 shows the average Pearson correlation coefficients between magnitude and phase from two different ROI signals across all the subjects in each group. The magnitude–phase correlation coefficients range from −0.2 to 0.2. This supports the magnitude–phase causality considered in the proposed method. Specifically, CER-related connections are marked with black boxes and locally magnified. There are polarity and strength differences between HCs and SZs in both magnitude–phase and phase–magnitude correlation coefficients. This suggests that the proposed complex-valued transfer entropy considering causality between magnitude–phase and phase–magnitude is essential and can capture more intergroup differences.

CER-related connections show more and higher significant intergroup differences obtained by the proposed CTE. Although the cerebellum has been reported to be associated with the motor system, a growing number of studies have found that the cerebellum is critical to processing complex functions, e.g., attention, cognition, and language (Lungu et al., 2013). Lungu et al. reviewed 234 fMRI studies published from 1997 to 2010 related to SZs and pointed out that 41.02% of the articles reported cerebellar activity related to cognitive, emotional, and executive processes in schizophrenia. In conclusion, the results of their analyses suggest that the cerebellum plays an essential functional role in schizophrenia, especially in the cognitive and executive domains. Following this, we performed searches in the abstracts of articles indexed in Scopus from 2011 to 2023 and found 218 articles that reported abnormal cerebellum-related connections in schizophrenia. These studies proved that the cerebellum is a functional hub involved in cognition, language, and emotional processing with regions, including TEM, DMN, and visual areas. For instance, Table 2 highlights Cerebelum_6_R (AAL No.100) has connections with significant HC-SZ difference, which is also consistent with previous studies. Su et al. (2013) quantified non-linear undirected connections and pointed out that cerebellum-related ROIs, especially CRBL6.R, were important in identifying schizophrenia. Zhuo et al. (2018) calculated FC density to investigate cerebellar connectivity changes of SZs and found abnormal connectivity strength of Cerebelum_6_R with visual areas (Zhuo et al., 2018).

Apart from CER, CTE also detects common connections related to visual areas (MV and LV), and temporal lobe (TEM) in Table 2. These two nodes have brain functions of vision and auditory, respectively. As hallucinations are a frequent symptom of schizophrenia including visual and auditory hallucinations occupying 70% of patients with schizophrenia (Demirci et al., 2008), it is expected that MV and TEM are schizophrenia-related in terms of pathology mechanisms (Fogelson et al., 2014; Dietz et al., 2020). For unique connections detected by CTE in Table 3, abnormal connectivity mainly related to RFP is verified by previous studies. Frontal parietal regions have been shown involved in the cognitive and perceptive process (Smith et al., 2009) and are highly related to the impaired cognitive function of SZs (Roiser et al., 2013). Roiser et al. pointed out that connective abnormality related to the frontal–parietal areas may link to cognitive impairment for SZs (Roiser et al., 2013), given that the unique abnormal connectivity patterns obtained by CTE may provide additional evidence for the cognitive and perceptive impairments of schizophrenia.

In addition to FC between ROI, CTE can also measure the FNC of brain networks. We use CTE to quantify the FNC of brain networks. Table 4 shows the SVM performance of the five directed analysis methods. Similar to FC results, CTE also shows the best performance among these methods. Compared with other directed analysis methods, CTE shows better classification performance, e.g., improves higher accuracy with 5.1% (88.2% vs. 83.1%) to sCTE, 9.9% (88.2% vs. 78.3%) to STE, 17.5% (88.2% vs. 70.7%) to HTE, and 16.9% (88.2% vs. 71.3%) to HTE, respectively.

**Table 4:** SVM classification is performed based on the directed FNC of brain networks.

	Accuracy (%)	Sensitivity (%)	Specificity (%)
CTE	88.2 ± 7.6	89.5 ± 4.8	86.9 ± 8.2
sCTE	83.1 ± 8.1	88.4 ± 6.7	77.8 ± 11.5
STE	78.3 ± 4.4	83.7 ± 7.1	72.9 ± 6.7
HTE	70.7 ± 7.8	78.0 ± 6.8	63.4 ± 5.9
Granger	71.3 ± 8.6	80.5 ± 4.9	62.1 ± 8.1

In future, our CTE approach can be extended to analyzing causal FNC of time courses extracted by blind source separation, e.g., ICA, sparse representation, and tensor decomposition. Second, dynamic-directed FC/FNC can be performed to further improve classification performance. Finally, CTE can be exploited for other mental disorders such as depressive disorder or further extended to other applications for evaluating complex-valued causality.

## Data availability statement

The original contributions presented in the study are included in the article/supplementary material, further inquiries can be directed to the corresponding authors.

## Ethics statement

The studies involving humans were approved by University of New Mexico Institutional Review Board. The studies were conducted in accordance with the local legislation and institutional requirements. The participants provided their written informed consent to participate in this study. Written informed consent was obtained from the individual(s) for the publication of any potentially identifiable images or data included in this article.

## Author contributions

W-XL: Conceptualization, Investigation, Methodology, Software, Writing – original draft, Writing – review & editing. Q-HL: Funding acquisition, Validation, Writing – review & editing. C-YZ: Methodology, Validation, Writing – review & editing. YH: Conceptualization, Validation, Writing – review & editing. VC: Data curation, Funding acquisition, Investigation, Validation, Writing – review & editing.

## Footnotes

^1^Available at: http://www.fil.ion.ucl.ac.uk/spm.
